# Spectrum of Abnormal Uterine Bleeding in the Women of Sub-Himalayan Hilly Region of North India: Clinicodemographic Profile and Management Options

**DOI:** 10.7759/cureus.102544

**Published:** 2026-01-29

**Authors:** Harpreet Kaur, Asmita Kaundal, Bhavna Bhavna, Nisha Malik, Sushruti Kaushal, Compal Chauhan

**Affiliations:** 1 Obstetrics and Gynecology, All India Institute of Medical Sciences, Bilaspur, Bilaspur, IND

**Keywords:** abnormal uterine bleeding, aub, dysfunctional uterine bleeding, menorrhagia, menstrual disturbances

## Abstract

Introduction

Abnormal uterine bleeding (AUB) is one of the common complaints affecting women across different stages of life.

Aim

The aim of this study was to determine the AUB spectrum in our population and correlate it with the histopathological spectrum.

Material and methods

This is a hospital-based prospective cross-sectional study of women presenting with AUB. The baseline demographic data and clinical details of menstrual complaints were collected through a pre-validated, semi-structured proforma after taking informed consent. Endometrial biopsy was offered to all eligible women for histopathological confirmation of diagnosis and ruling out malignancy.

Results

A total of 260 women were enrolled. Heavy menstrual bleeding was the most common complaint (56.2%), followed by prolonged bleeding (16.9%), and irregular bleeding (18.4%). The histopathological results confirmed the diagnosis of secretory endometrium in 17.3%, proliferative endometrium in 49.2%, disordered proliferative endometrium in 25.4%, simple hyperplasia in 3.5%, endometrial polyp in 3.5%, and endometrial carcinoma in 1.2%. Majority of the women were classified as AUB-N (36.2%) according to the PALM-COEIN classification. Majority received medical management, either antifibrinolytic (98.5%), non-hormonal therapy (0.8%), or hormonal therapy in the form of oral progesterone (16.9%), combined oral contraceptive pills (1.9%), and levonorgestrel-releasing intrauterine contraceptive device (0.8%). Surgical management in the form of hysterectomy (20.8%) and hysteroscopy-guided endometrial polypectomy (1.9%) was also offered.

Conclusion

AUB affects women across all age groups, significantly affecting their quality of life. AUB-N, AUB-O and AUB-L contribute to a major proportion of AUB. A detailed evaluation, including histopathology, is required to rule out the associated cause and malignant/pre- malignant lesions of the endometrium.

## Introduction

Despite advancements in the medical field and the availability of affordable treatment, AUB still remains a major gynecological condition affecting women of all age groups, starting from adolescence to menopause. AUB may lead to significant physical and social burden and impaired quality of life for women in the most productive years of life [1-2]. In a non-pregnant woman, any deviation from a normal menstrual pattern is termed AUB. The deviation can be in regularity, frequency, duration, or amount of blood loss during the menstrual cycles. AUB can be classified as acute or chronic based on presenting symptoms and symptom duration. Acute AUB is when a woman presents with abnormal bleeding requiring immediate medical or surgical intervention to control bleeding [3]. However, AUB is termed chronic when the symptoms are present for at least six months. Acute AUB may often be superimposed on chronic AUB. Women in the perimenopausal age group more commonly experience alterations in the menstrual cycle. To standardize the nomenclature of AUB, the International Federation of Gynaecology and Obstetrics (FIGO) introduced a new system known by the acronym PLAM-COEIN (Polyp, Adenomyosis, Leiomyoma, Malignancy and hyperplasia, Coagulopathy, Ovulatory disorders, Endometrial factors, Iatrogenic, and Not classified) in 2011. Also, “amenorrhea” is defined as the absence of menstrual bleeding during a six-month reference period. The PALM-COEIN system is etiopathogenesis-based, with PALM describing structural causes and COEIN demoting non-structural causes of AUB [4-5]. A large proportion of women presenting to our gynecology outpatient department (OPD) had complaints of abnormal bleeding. This prompted us to conduct this study to know the spectrum of AUB in our locality. Understanding the clinical profile of women with AUB in the local population is important for identifying the common causes of the condition and, accordingly, formulating interventions aimed at preventing it. It is equally important to know the etiology and histopathology of women with AUB and their management options.

We planned this with the objective to determine the demographic and clinical profiles of women presenting with AUB and to evaluate their spectrum of histopathology findings and management options.

## Materials and methods

A total of 260 women who presented to the gynecological OPD with abnormal uterine bleeding from March 2023 to March 2024 were included in this hospital-based prospective cross-sectional study after attaining the ethical committee approval (IEC number: 27/22 dated 10/03/2023).

Sample size

The sample size was calculated to be 226 at a 95% confidence interval and a relative precision of 5%, considering the reported prevalence of AUB as 17.9% in Indian females with Epi-Info [6].

However, a total of 273 women were enrolled in the study initially, of whom 13 patients were lost as they did not follow up either for endometrial sampling or for continuing their follow-up; the data of these women were not used in the final calculation, and the data of 260 women was finally evaluated for final results.

Study population

The study was conducted on women above the age of 18 years, presenting to the gynecology OPD with complaints of menstrual abnormalities. Women less than 18 years of age, with known congenital anomalies of the uterus/genital tract, pregnant women, post-partum or post-abortal women (up to 42 days after pregnancy), lactating women, those who had already undergone hysterectomy, and those not willing to participate in the study/follow-up were not included in the study.

After obtaining written informed consent, a pre-validated, semi-structured proforma was used to collect the demographic and clinical details from women complaining of menstrual abnormality. Detailed information regarding the demographic profile, type of menstrual abnormality, type of treatment sought, investigations done, and the management options discussed was recorded by the investigator. In addition, a discussion was carried out regarding different treatment options, including medical/hormonal/surgical treatment, depending on the etiology of AUB. Initial investigations included a complete blood count, urine pregnancy test, thyroid-stimulating hormone (TSH), blood sugar levels, and a transvaginal ultrasonography. In cases of thickened endometrium, an endometrial biopsy was taken and sent for histopathological examination. Based on the clinical or histopathological diagnosis, participant women were explained in detail regarding medical (hormonal and non-hormonal) and surgical management after explaining the risks versus advantages of all the available modalities.

Outcomes

The primary outcome measured was to determine the demographic and clinical profile of women presenting with abnormal uterine bleeding. The secondary outcome was evaluation of the spectrum of histopathology findings and management options for women with AUB.

Statistical analysis

Descriptive and inferential statistical analyses were conducted. Results on continuous measurements were presented as mean ± SD (min-max), and results on categorical measurements were presented as number (%). Significance was assessed at 5% level of significance. The following assumptions on data were made: (1) dependent variables should be normally distributed and (2) samples drawn from the population should be random, and cases of the samples should be independent.

The one-way analysis of variance (ANOVA) was employed to determine whether there were any statistically significant differences between the means of three or more independent (unrelated) groups. The one-way ANOVA compares the means between the groups you are interested in and determines whether any of those means are statistically significantly different from each other. Specifically, it tests the null hypothesis: Ho:µ1=µ2=µ3=....=µk, where µ = group mean and k = number of groups. If, however, the one-way ANOVA returns a statistically significant result, we accept the alternative hypothesis (HA), which is that there are at least two group means that are statistically significantly different from each other.

Assumptions for ANOVA test are as follows: (1) the dependent variable is normally distributed in each group that is being compared in the one-way ANOVA; (2) there is homogeneity of variances, meaning that the population variances in each group are equal; (3) independence of observations.

Fisher’s exact test was used to find the significance of study parameters on a categorical scale between two or more groups, and non-parametric setting was used for qualitative data analysis. Fisher's exact test is used when cell samples are very small.

A p-value of <0.05 was considered significant. SPSS Version 16.0 (IBM Corp., Armonk, NY) was used for analysis.

## Results

The final analysis was conducted on data collected from a total of 260 women. Table 1 summarizes the basic characteristics of study participants. The mean age of the study participants was 43.95±7.75 SD years, and the majority (56.9%) were between the age groups of 41-50 years. Most women were from rural backgrounds, 227/260 (87.3%), and were unemployed, 33/260 (88.5%). More than a third of women, 101/260 (38.8%), were in the pre-obese category with a BMI between 25 and 29.9 kg/m^2^, 46/260 (17.7%) were obese with a BMI of >30 kg/m^2^, and 35/260 (13.5%) were overweight with a BMI of 7/260 (2.7%) were underweight for age. Most of the women, 234/260 (90%), were multiparous. One or more comorbid conditions was present in 64/260 (24.6%) of the women, with the commonest comorbidities being hypertension, 23/260 (8.8%), and diabetes mellitus, 8/260 (3.1%). The mean hemoglobin level of the study participants was 10.73±1.7 g/dL, platelet count was 3.42±16.12 thousand/mm^3^, and TSH level was 3.95±13.17 µIU/mL.

**Table 1:** Baseline demographic data of the study population (N=260) The table shows the baseline demographic data, including age, BMI, education, occupation, parity, and comorbid conditions of the study population.

Variables	No. of Patients (n=260)	%	Mean + SD
Age (years)			
<20	3	1.2	43.95+7.75
21-30	15	5.8	
31-40	54	20.8	
41-50	148	56.9	
>51	40	15.4	
BMI category (kg/m^2^)			
Underweight	7	2.7	28.1+5.7
Normal	71	27.3	
Pre-obese	101	38.8	
Obese	46	17.7	
Overweight	35	13.5	
Residence			
Rural	227	87.3	-
Urban	33	12.7	
Education			
Illiterate	14	5.4	-
Primary	77	29.6	
Secondary	51	19.6	
Senior secondary	58	22.3	
Graduate	38	14.6	
Postgraduate	22	8.5	
Parity			
Nulliparous	19	7.3	-
Primiparous	7	2.7	
Multiparous	234	90.0	
Occupation			
Employed	30	11.5	-
Unemployed	230	88.5	
Comorbid illness			
Nil	196	75.4	-
Yes	64	24.6	
Hypertension	23	8.8	
Diabetes mellitus	8	3.1	
Asthma	4	1.5	
Epilepsy	2	0.8	
Migraine	2	0.8	
Cholecystectomy	2	0.8	
Others	23	8.8	

**Figure 1:**
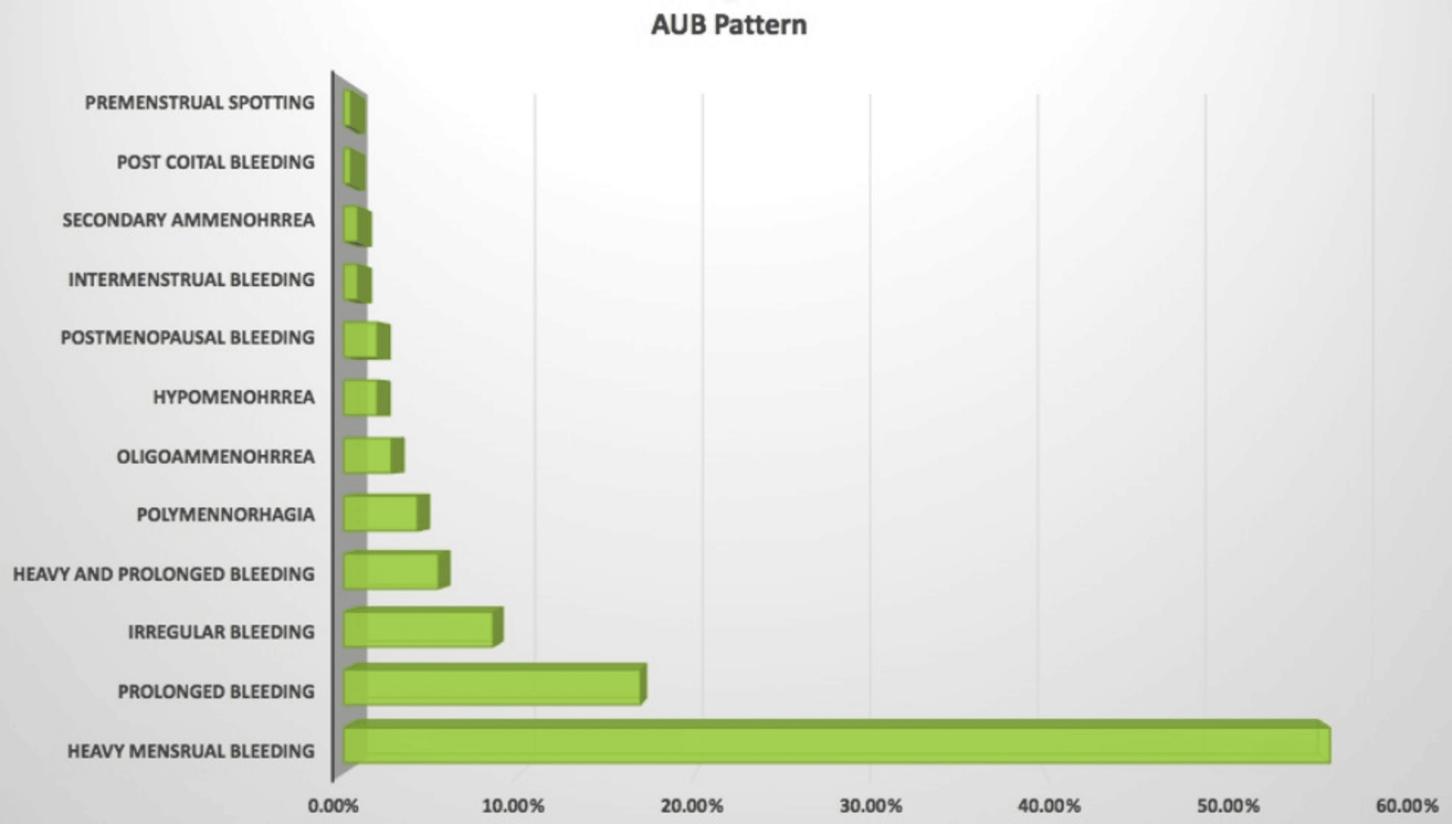
AUB spectrum of presenting symptoms The figure illustrates the distribution of the bleeding pattern in the study population. Values are expressed in percentage of the total number of participants. AUB, abnormal uterine bleeding

Figure 1 depicts the patterns of abnormal uterine bleeding with which women presented to the OPD. Out of all, HMB was the most common complaint (56.2%), followed by prolonged bleeding, 44/260 (16.9%), or irregular bleeding, 22 (8.5%). Dysmenorrhea was also present in nearly two-thirds of the participants, along with AUB, 194/260 (74.6%).

On examination, 5/260 (1.9%) of participants presented with a large abdominal pelvic mass of 14-16 weeks in size. The size of the uterus varied between 10 and 12 weeks in 23/260 (8.8%) of women, 8 and 10 weeks in 20 (7.7%), and 6 and 8 weeks in 20/260 (7.7%). Adnexal mass was present in 46/260 (17.7%) women and cervical polyps in 5/260 (1.9%). On ultrasound, endometriotic cysts were found in 30/260 (11.5%), leiomyoma in 55/260 (21.2%), adenomyosis in 17/260 (6.5), simple ovarian cyst in 16/260 (6.2%), and endometrial polyp in 09/260 (3.5%) patients. Thickened endometrium with endometrial thickness of >12mm was found in 145/260 (55.8%) women. The histopathological results from the endometrial sample revealed a diagnosis of proliferative endometrium in 128/260 (49.2%), disordered proliferative endometrium in 66/260 (25.4%), secretory endometrium in 45/260 (17.3%), simple hyperplasia in 09/260 (3.5%), endometrial polyp in 09/260 (3.5%), and endometrial carcinoma 03/260 (1.2%) (Table 2).

**Table 2:** Histopathological pattern in women with AUB (N=260) The table describes the various histopathological patterns of the endometrial biopsy reports in the study population. AUB, abnormal uterine bleeding

Histopathological pattern	(N=260)	% age
Proliferative endometrium	128	49.2%
Disordered proliferative endometrium	66	25.4%
Secretory endometrium	45	17.3%
Simple hyperplasia	9	3.5%
Endometrial polyp	9	3.5%
Endometrial carcinoma	3	1.2%

As per the PALM-COEIN classification, PALM constituted 93/260 (35.8%) and COEIN 167/260 (64.2%).

**Figure 2:**
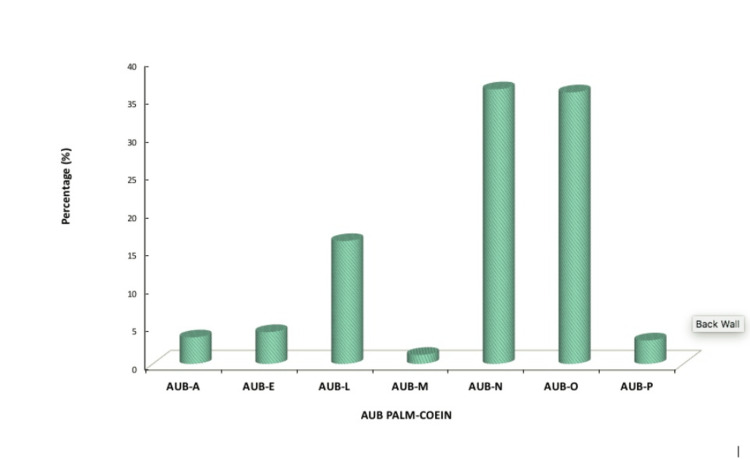
AUB pattern as per the PALM-COEIN classification The figure depicts the AUB pattern as per PALM-COEIN classification: Polyp, Adenomyosis, Leiomyoma, Malignancy and hyperplasia, Coagulopathy, Ovulatory dysfunction, Endometrial, Iatrogenic, and Not classified. The number of patients is represented in percentage. AUB, abnormal uterine bleeding

Figure 2 depicts the distribution of the AUB pattern as per PALM-COEIN classification. Most of the women were diagnosed as AUB-N, 94/260 (36.2%). Almost all the women were initially treated with antifibrinolytic, 256/260 (98.5%). Hormonal therapy in the form of oral progesterone was given to 44/260 (16.9%), combined oral contraceptive pills to 5/260 (1.9%), and levonorgestrel-releasing intrauterine contraceptive device to 2/260 (0.8%) of women, and a selective estrogen receptor modulator (tablet Ormeloxifene) to 2/260 (0.8%). Around 59/260 (22.7%) women resorted to surgery, with hysterectomy being performed in 54/260 (20.8%) of women and hysteroscopic-guided polypectomy for endometrial polyps in 5/260 (1.9%).

When we correlated the AUB pattern with different variables, no significant correlation was seen between the various demographic variables (age, BMI, residence, comorbid conditions, hemoglobin level, platelet count, and TSH level (Table 3).

**Table 3:** Correlation between clinical variables and AUB pattern in the study participants The table depicts the correlation between the AUB pattern and clinical variables like age, BMI, urban or rural status, parity, comorbid conditions, hemoglobin level, platelet count, and TSH level. A p-value of <0.05 is taken as significant. Fisher’s exact test was applied. AUB, abnormal uterine bleeding; TSH, thyroid-stimulating hormone

Variables	Diagnosis							Total	P-value
	AUB-A	AUB-E	AUB-L	AUB-M	AUB-N	AUB-O	AUB-P		
Age in years									
<20	0 (0%)	0 (0%)	0 (0%)	0 (0%)	1 (1.1%)	2 (2.2%)	0 (0%)	3 (1.2%)	0.147
20-30	0 (0%)	1 (9.1%)	0 (0%)	0 (0%)	8 (8.5%)	6 (6.5%)	0 (0%)	15 (5.8%)	
32-40	4 (44.4%)	1 (9.1%)	9 (21.4%)	0 (0%)	15 (16%)	23 (24.7%)	2 (25%)	54 (20.8%)	
41-50	4 (44.4%)	8 (72.7%)	26 (61.9%)	0 (0%)	53 (56.4%)	52 (55.9%)	5 (62.5%)	148 (56.9%)	
>50	1 (11.1%)	1 (9.1%)	7 (16.7%)	3 (100%)	17 (18.1%)	10 (10.8%)	1 (12.5%)	40 (15.4%)	
Residence									
Rural	9 (100%)	9 (81.8%)	35 (83.3%)	2 (66.7%)	86 (91.5%)	80 (86%)	6 (75%)	227 (87.3%)	0.414
Urban	0 (0%)	2 (18.2%)	7 (16.7%)	1 (33.3%)	8 (8.5%)	13 (14%)	2 (25%)	33 (12.7%)	
Body mass index									
Underweight	0 (0%)	0 (0%)	1 (2.4%)	0 (0%)	3 (3.2%)	3 (3.2%)	0 (0%)	7 (2.7%)	0.772
Normal	2 (22.2%)	2 (18.2%)	7 (16.7%)	0 (0%)	35 (37.2%)	23 (24.7%)	2 (25%)	71 (27.3%)	
Pre-obese	4 (44.4%)	7 (63.6%)	17 (40.5%)	2 (66.7%)	29 (30.9%)	39 (41.9%)	3 (37.5%)	101 (38.8%)	
Obese	1 (11.1%)	2 (18.2%)	10 (23.8%)	1 (33.3%)	13 (13.8%)	18 (19.4%)	1 (12.5%)	46 (17.7%)	
Overweight	2 (22.2%)	0 (0%)	7 (16.7%)	0 (0%)	14 (14.9%)	10 (10.8%)	2 (25%)	35 (13.5%)	
Parity									
Nulliparous	0 (0%)	0 (0%)	3 (7.1%)	0 (0%)	10 (10.6%)	6 (6.5%)	0 (0%)	19 (7.3%)	0.905
Primiparous	0 (0%)	0 (0%)	1 (2.4%)	0 (0%)	4 (4.3%)	2 (2.2%)	0 (0%)	7 (2.7%)	
Multiparous	9 (100%)	11 (100%)	38 (90.5%)	3 (100%)	80 (85.1%)	85 (91.4%)	8 (100%)	234 (90%)	
Comorbid illness									
Nil	7 (77.8%)	8 (72.7%)	32 (76.2%)	0 (0%)	73 (77.7%)	72 (77.4%)	4 (50%)	196 (75.4%)	0.051
Yes	2 (22.2%)	3 (27.3%)	10 (23.8%)	3 (100%)	21 (22.3%)	21 (22.6%)	4 (50%)	64 (24.6%)	
Hemoglobin (g/dL)	10.62±1.23	11.11±1.56	10.82±1.66	12.13±0.42	10.91±1.85	10.51±1.56	9.86±2.03	10.73±1.7	0.276
Platelets	1.35±0.57	1.94±0.55	6.4±27.93	2.18±0.19	4.03±19.29	2±0.63	1.97±0.66	3.42±16.12	0.859
TSH (µIU/mL)	2.74±1.28	2.72±0.72	2.95±1.81	3.08±2.18	5.83±21.74	2.81±1.62	3.77±1.76	3.95±13.17	0.807

## Discussion

Any abnormality in the menstrual cycles outside pregnancy is termed AUB. The abnormality can be related to frequency (<24 days or >38 days), duration (< 4.5 days or > 8 days), volume (<5mL or >80mL/day), and regularity (absent cycles or variation of >20 days). Abnormal uterine bleeding is one of the most common gynecological conditions, with the incidence varying between 14% and 25% across the globe [7-8]. It not only causes poor health but also affects mental health and social life.

 AUB is mostly seen around the menarche or in the perimenopausal age group and often increases with advancing age [9]. In the present study, we have seen that most of the women (93.1%) who presented with AUB were >30 years of age, with a predominance (56.9%) between 41 and 50 years. Similar results were found in the previous study conducted by Singh and Sonawane, where the authors reported that around 68.7% of the cases presented after 30 years of age [10]. Another study conducted by Singh et al. to study the spectrum of endometrial pathology in AUB also reported that the majority of the histopathological specimens were from women between the age group of 40-59 years (66.5%) [9]. In our study, most of the women who presented with AUB were multiparous (>1 childbirth) (90%). A study conducted by Karimi et al. and Talukdar and Mahela also reported similar results, where around 58% of women presenting with AUB were multiparous [11-12].

We found that most of the women presented with symptoms of HMB (56.2%). The results were similar to previously conducted studies by Karimi et al., where 48% of women presented with HMB. Kumari et al. also found that HMB was the most common presentation of AUB in their study population, followed by light menstrual bleeding, irregular cycles, no bleeding, frequent cycles, and infrequent cycles [13]. However, a study conducted by Bindroo et al. found that premenopausal bleeding (86.4%) was the most common presentation in their study group, followed by postmenopausal bleeding (13.6%) [14].

We found that that most of the cases (36.2%) were attributed to AUB-N (not otherwise classified), followed by AUB-O (ovulatory dysfunction) (35.8%) and AUB-L (leiomyoma) (16.2%). Singh et al. reported that the majority of the cases (55.2%) were because of ovulatory dysfunction (AUB-O), followed by leiomyoma (AUB-L) [9]. Another study conducted by Kumari et al. also found that AUB-O was the most common cause of AUB in the study, followed by AUB-L, AUB-A, AUB-P, AUB-M, AUB-E, and AUB-C.

FIGO recommends ruling out premalignant and malignant lesions by histopathological examination of the endometrial tissue in perimenopausal women and those at high risk for endometrial cancers. Endometrial biopsy is recommended for women presenting with AUB, those above 40 years of age, and those less than 40 years of age with other high-risk factors for endometrial carcinoma.

Eligible candidates in the present study also underwent endometrial aspiration, and the tissue obtained was sent for histopathological examination. The most common type of histopathological finding was proliferative endometrium (49.2%). Most previous studies reported proliferative endometrium as the most common histopathological finding in the endometrial specimen, followed by secretory, disordered proliferative endometrium, and endometrial hyperplasia. Like our study, Bindroo et al. reported a proliferative endometrium pattern (37.2%). Similar to the study conducted by Abid et al., we also found secretory endometrium as the most common histopathological pattern, followed by a proliferative pattern [15].

Limitations of the study

The study is a single-center study conducted in a small group of patients. More studies on a larger population are needed to understand the exact burden of the condition and the histopathological correlation.

## Conclusions

AUB is a major gynecological condition commonly affecting women in the perimenopausal age group; however, the disease can affect women at all stages of life, starting from menarche to post-menopause. Based on our study, HMB is the most common presentation and is seen mostly in perimenopausal women. Detailed evaluation through clinical history, examination, and ultrasound is essential to find the cause. Endometrial sampling for histopathology is essential to differentiate between benign and malignant conditions.
